# *catena*-Poly[(μ_3_-2-hy­droxy-4-isopropyl­cyclo­hepta-2,4,6-trien-1-onato)(μ_2_-2-hy­droxy-4-isopropyl­cyclo­hepta-2,4,6-trien-1-onato)lead(II)]

**DOI:** 10.1107/S1600536810039978

**Published:** 2010-10-13

**Authors:** Krzysztof Lyczko, Monika Lyczko, Wojciech Starosta

**Affiliations:** a Institute of Nuclear Chemistry and Technology, Dorodna 16, 03-195 Warsaw, Poland

## Abstract

In the title compound, [Pb(C_10_H_11_O_2_)_2_]_*n*_ or [Pb(hino)_2_]_*n*_, the lead(II) ion is chelated by two hinokitiolate ligands in a distorted square-pyramidal configuration, with Pb—O bond lengths in the range 2.327 (6)–2.479 (9) Å. The 6*s* ^2^ lone electron pair of the lead(II) ion becomes stereochemically active and is directed towards the apex of this pyramid. The crystal structure of the title compound consists of chains formed by the bis­(hinokitiolato)lead(II) mol­ecules situated along the twofold screw axis. The coordination sphere around the lead(II) ion is completed by three additional O atoms, at 2.625 (7), 3.016 (8) and 3.064 (8) Å, from the two neighbouring Pb(hino)_2_ units. Both isopropyl groups are rotationally disordered.

## Related literature

For structural data on hinokitiolato–metal complexes, see: Abrahams *et al.* (1994 ▶); Barret *et al.* (2000 ▶, 2001 ▶, 2002 ▶); Nomiya *et al.* (2004 ▶, 2009 ▶); Ho (2010 ▶). For related structures, see: Malik *et al.* (1999 ▶); Harrowfield *et al.* (2004 ▶); Lyczko *et al.* (2006 ▶, 2007 ▶). For hemi- and holodirected geometries of lead(II) complexes, see: Shimoni-Livny *et al.* (1998 ▶). For the van der Waals radii of lead and oxygen, see: Bondi (1964 ▶).
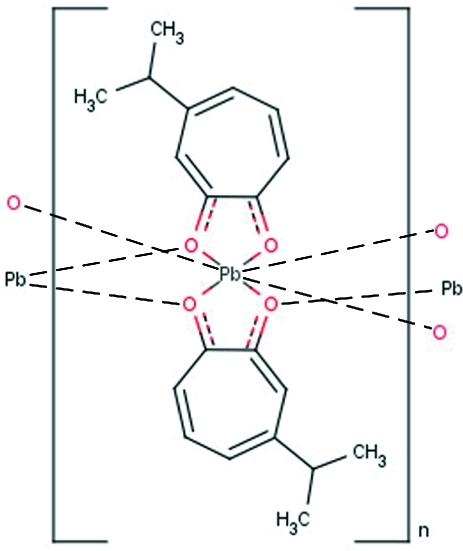


## Experimental

### 

#### Crystal data

[Pb(C_10_H_11_O_2_)_2_]
*M* *_r_* = 533.57
Orthorhombic, 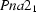

*a* = 33.780 (7) Å
*b* = 8.2802 (17) Å
*c* = 7.3661 (15) Å
*V* = 2060.3 (7) Å^3^
*Z* = 4
Mo *K*α radiation
μ = 8.21 mm^−1^
*T* = 293 K
0.35 × 0.05 × 0.03 mm

#### Data collection

Kuma KM-4 four-circle diffractometer
Absorption correction: analytical (*CrysAlis RED*; Oxford Diffraction, 2000 ▶) *T*_min_ = 0.527, *T*_max_ = 0.789
3452 measured reflections
3063 independent reflections
1969 reflections with *I* > 2σ(*I*)
*R*_int_ = 0.093
3 standard reflections every 200 reflections  intensity decay: 1.1%

#### Refinement

*R*[*F* ^2^ > 2σ(*F* ^2^)] = 0.032
*wR*(*F* ^2^) = 0.093
*S* = 1.06
3063 reflections
216 parameters
13 restraints
H-atom parameters constrained
Δρ_max_ = 1.38 e Å^−3^
Δρ_min_ = −2.11 e Å^−3^

### 

Data collection: *KM-4 Software* (Kuma, 1996 ▶); cell refinement: *KM-4 Software*; data reduction: *DATAPROC* (Kuma, 2001 ▶); program(s) used to solve structure: *SHELXS97* (Sheldrick, 2008 ▶); program(s) used to refine structure: *SHELXL97* (Sheldrick, 2008 ▶); molecular graphics: *XP* in *SHELXTL* (Sheldrick, 2008 ▶); software used to prepare material for publication: *SHELXL97*.

## Supplementary Material

Crystal structure: contains datablocks global, I. DOI: 10.1107/S1600536810039978/bt5353sup1.cif

Structure factors: contains datablocks I. DOI: 10.1107/S1600536810039978/bt5353Isup2.hkl

Additional supplementary materials:  crystallographic information; 3D view; checkCIF report

## Figures and Tables

**Table 1:** Selected bond lengths (Å)

Pb1—O11	2.327 (6)
Pb1—O12	2.420 (7)
Pb1—O1	2.422 (7)
Pb1—O2	2.479 (9)
Pb1—O12^i^	2.625 (7)
Pb1—O1^i^	3.016 (8)
Pb1—O11^ii^	3.064 (8)

Symmetry codes: (i) 
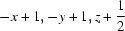
; (ii) 
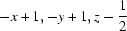
.

**Fig. 1.**
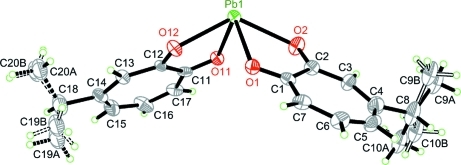
Molecular structure of the title compound with disordered methyl C atoms of isopropyl groups. Displacement ellipsoids of the non-hydrogen atoms are drawn at the 30% probability level.

**Fig. 2.**
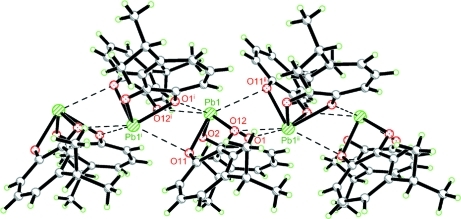
A fragment of the crystal structure of Pb(hino)2. The additional weak Pb···O contacts shown with dashed line. [Symmetry codes: (i) -x + 1, -y + 1, z + 1/2; (ii) -x + 1, -y + 1, z - 1/2].

## supplementary crystallographic information

### Comment

Hinokitiol (2-hydroxy-4-isopropyl-2,4,6-cycloheptatrien-1-one), also called β-thujaplicin, abbreviated as Hhino, is a derivative of tropolone (Htrop) with isopropyl group in the position 4 of the aromatic seven-membered carbon ring. The hinokitiolate (hino^-^) anion, like the tropolonate (trop^-^) anion, is a bidentate oxygen donor ligand, which forms five-membered ring upon complexation to metal ions.

The structural data on metal complexes with hinokitiol are limited to only several examples. There are a few structures of homoleptic hinokitiolato-metal complexes such as [Cu(hino)_2_] (two forms, *cis* and *trans*, have been structurally characterized; Barret *et al.*, 2002), [Pd(hino)_2_], [Sb(hino)_3_], [Zr(hino)_4_] (Nomiya *et al.*, 2009), [In(hino)_3_] (Abrahams *et al.*, 1994) and [Al(hino)_3_] (Nomiya *et al.*, 2004). Moreover, some dimeric species, *e.g.* [Ag(hino)]_2_, [*M*(hino)_2_(EtOH)]_2_ (*M* = Co, Zn, Ni, Mg) (Nomiya *et al.*, 2004, 2009; Barret *et al.*, 2001), [Mn(hino)_2_(MeOH)]_2_ and [Bi(hino)_3_(EtOH)]_2_ (Nomiya *et al.*, 2009), and one trimeric [Cu(hino)_2_]_3_ (Ho, 2010) have been reported. A small number of hinokitiolato-metal complexes with mixed ligands, *e.g.*, [Sn*X*_2_(hino)_2_] (*X* = F, Cl) (Barret *et al.*, 2000) and [MoO_2_(hino)_2_] (Nomiya *et al.*, 2009) have been structurally characterized.

The aim of this work was to determine the structure of the homoleptic complex formed by the lead(II) cation and hinokitiolate anions.

The molecular structure of the title compound consists of the lead(II) ion chelated by two hino^-^ ions (Fig. 1). The lead atom and four oxygen atoms form a distorted square pyramid with Pb—O bond lengths of 2.327 (6), 2.420 (7), 2.422 (7) and 2.479 (9) Å, mean 2.412 Å. The bite angles in the chelate are equal to 64.1 (2) and 67.3 (2)°.

The square pyramidal configuration around metal ion (hemidirected geometry; Shimoni-Livny *et al.*, 1998), is an evidence for the presence of a stereochemically active 6*s*^2^ lone electron pair of the lead(II) ion at the apex of this pyramid, like in bis(tropolonato)lead(II) (Lyczko *et al.*, 2007) and bis(β-diketonato)lead(II) complexes (Malik *et al.*, 1999; Harrowfield *et al.*, 2004; Lyczko *et al.*, 2006;).

The crystal structure of the studied compound consists of chains parallel to the *c* axis, formed by the Pb(hino)_2_ molecules situated around the twofold screw axis. The coordination sphere of the lead(II) ion can be completed by three additional bridging oxygen atoms from the adjacent ligands of two neighbouring Pb(hino)_2_ molecules (Fig. 2). These three Pb···O contacts, one rather short, 2.625 (7), and two more distant, 3.016 (8) and 3.064 (8) Å, are much longer than the Pb—O bonds in the chelate, but shorter than the sum of van der Waals radii of lead and oxygen (3.44 Å; Bondi, 1964), which points out to weak interactions. The additional Pb···O distances in the Pb(hino)_2_ structure lead to a formal increase of the coordination number of the lead(II) ion from four to seven.

A similar crystal structure with a bit longer additional Pb···O contacts at 3.013 (5), 3.159 (6) and 3.264 (6) Å was found for bis(acetylacetonato)lead(II), Pb(acac)_2_ (Lyczko *et al.*, 2006). The crystal packing in the title compound significantly differs from that obtained for bis(tropolonato)lead(II) complex (Lyczko *et al.*, 2007), in which the [Pb(trop)_2_]_2_ dimeric units can be observed. Nevertheless the main structural feature, which is the distorted square pyramid, looks very similar in all lead(II) α- or β-diketonates discussed above.

### Experimental

Lead(II) acetate trihydrate (0.188 g, 0.496 mmol) was dissolved in water (1.5 ml). A methanol solution (1.0 ml) of hinokitiol (0.171 g, 1.041 mmol) was added to the aqueous solution and the precipitation of the title compound took place. The bis(hinokitiolato)lead(II) compound was filtrated off and washed with methanol and acetone. By slow diffusion of n-hexane into a dichloromethane solution of the studied complex dark yellow crystals were obtained. Elemental analysis calculated for C_20_H_22_O_4_Pb: C 45.02, H 4.16%; found: C 44.86, H 4.15%. IR (KBr): 3035 (vw), 2959 (*m*), 2928 (vw), 2867 (vw), 1587 (*s*), 1497 (*s*), 1449 (w), 1442 (*versus*), 1383 (vw), 1364 (*s*), 296 (vw), 1239 (*m*), 1187 (vw), 960 (w), 924 (vw), 910 (vw), 883 (vw), 805 (w), 773 (vw), 756 (vw), 734 (w), 667 (vw), 641 (vw), 523 (vw), 494 (w), 416 (vw) cm^-1^.

### Refinement

H atoms were placed in calculated positions with C—H = 0.96 (methyl), 0.98 (methine) or 0.93 Å (aromatic) and were refined isotropic using a riding model with *U*_iso_(H) = 1.5 *U*_eq_(C) for methyl H atoms and *U*_iso_(H) = 1.2 *U*_eq_(C) for the rest H atoms. The structure of studied complex fits very well to the collected data. However, the absolute structure could not be determined because Friedel pairs were not measured and in fact the obtained Flack parameter has no physical sense. The structure was treated with two independent rotational disorders originating from two different isopropyl groups. For the first C_3_H_7_ group two-site disorder model consisting of two sets of atoms: C9A, C10A and C9B, C10B with site occupancy factors of 0.69 (4) and 0.31 (4), respectively was refined. The similar model containing C19A, C20A and C19B, C20B atoms with sof of 0.60 (6) and 0.40 (6), respectively was found for the second C_3_H_7_ group. C8 and C18 are shared atoms for both components of the disorder in the respective isopropyl groups. Rigid bonds and equal ADPs restraints in the isopropyl parts were employed in the refinement process.

### Crystal data

**Table:**

[Pb(C_10_H_11_O_2_)_2_]	*F*(000) = 1024
*M**_r_* = 533.57	*D*_x_ = 1.720 Mg m^−^^3^
Orthorhombic, *P**n**a*2_1_	Mo *K*α radiation, λ = 0.71073 Å
Hall symbol: P 2c -2n	Cell parameters from 25 reflections
*a* = 33.780 (7) Å	θ = 6–15°
*b* = 8.2802 (17) Å	µ = 8.21 mm^−^^1^
*c* = 7.3661 (15) Å	*T* = 293 K
*V* = 2060.3 (7) Å^3^	Plate, dark yellow
*Z* = 4	0.35 × 0.05 × 0.03 mm

### Data collection

**Table:**

Kuma KM-4 four-circle diffractometer	1969 reflections with *I* > 2σ(*I*)
Radiation source: fine-focus sealed tube	*R*_int_ = 0.093
graphite	θ_max_ = 30.0°, θ_min_ = 1.2°
profile data from ω/2θ scan	*h* = 0→45
Absorption correction: analytical (*CrysAlis RED*; Oxford Diffraction, 2000)	*k* = −11→1
*T*_min_ = 0.527, *T*_max_ = 0.789	*l* = 0→10
3452 measured reflections	3 standard reflections every 200 reflections
3063 independent reflections	intensity decay: 1.1%

### Refinement

**Table:**

Refinement on *F*^2^	Secondary atom site location: difference Fourier map
Least-squares matrix: full	Hydrogen site location: inferred from neighbouring sites
*R*[*F*^2^ > 2σ(*F*^2^)] = 0.032	H-atom parameters constrained
*wR*(*F*^2^) = 0.093	*w* = 1/[σ^2^(*F*_o_^2^) + (0.0528*P*)^2^] where *P* = (*F*_o_^2^ + 2*F*_c_^2^)/3
*S* = 1.06	(Δ/σ)_max_ = 0.001
3063 reflections	Δρ_max_ = 1.38 e Å^−^^3^
216 parameters	Δρ_min_ = −2.11 e Å^−^^3^
13 restraints	Absolute structure: 0 Friedel pairs
Primary atom site location: structure-invariant direct methods	Flack parameter: −0.004 (18)

### Special details

**Table:**

Geometry. All e.s.d.'s (except the e.s.d. in the dihedral angle between two l.s. planes) are estimated using the full covariance matrix. The cell e.s.d.'s are taken into account individually in the estimation of e.s.d.'s in distances, angles and torsion angles; correlations between e.s.d.'s in cell parameters are only used when they are defined by crystal symmetry. An approximate (isotropic) treatment of cell e.s.d.'s is used for estimating e.s.d.'s involving l.s. planes.
Refinement. Refinement of *F*^2^ against ALL reflections. The weighted *R*-factor *wR* and goodness of fit *S* are based on *F*^2^, conventional *R*-factors *R* are based on *F*, with *F* set to zero for negative *F*^2^. The threshold expression of *F*^2^ > σ(*F*^2^) is used only for calculating *R*-factors(gt) *etc*. and is not relevant to the choice of reflections for refinement. *R*-factors based on *F*^2^ are statistically about twice as large as those based on *F*, and *R*- factors based on ALL data will be even larger.

### Fractional atomic coordinates and isotropic or equivalent isotropic displacement parameters (Å^2^)

**Table:**

	*x*	*y*	*z*	*U*_iso_*/*U*_eq_	Occ. (<1)
Pb1	0.481976 (9)	0.57161 (3)	0.75652 (10)	0.03247 (9)	
O1	0.4460 (2)	0.4808 (9)	0.4911 (10)	0.0418 (16)	
O11	0.4841 (2)	0.3176 (7)	0.8915 (10)	0.0380 (15)	
O12	0.5278 (2)	0.3903 (9)	0.6075 (9)	0.0383 (18)	
C6	0.3480 (4)	0.4735 (14)	0.285 (2)	0.051 (3)	
H6	0.3422	0.4691	0.1621	0.061*	
C7	0.3888 (4)	0.4763 (13)	0.3168 (15)	0.047 (3)	
H7	0.4050	0.4696	0.2149	0.056*	
C1	0.4082 (4)	0.4881 (11)	0.4871 (13)	0.037 (2)	
C2	0.3877 (4)	0.5141 (14)	0.6634 (14)	0.041 (3)	
C12	0.5365 (3)	0.2592 (10)	0.6953 (14)	0.029 (2)	
C11	0.5116 (3)	0.2171 (12)	0.8453 (13)	0.034 (2)	
C3	0.3461 (3)	0.5041 (16)	0.6895 (17)	0.050 (3)	
H3	0.3393	0.5113	0.8116	0.060*	
C5	0.3149 (4)	0.4757 (16)	0.388 (2)	0.062 (4)	
H5	0.2909	0.4695	0.3269	0.075*	
C4	0.3133 (4)	0.486 (2)	0.581 (2)	0.065 (4)	
O2	0.4095 (3)	0.5386 (11)	0.7952 (9)	0.054 (2)	
C17	0.5161 (4)	0.0757 (11)	0.9515 (17)	0.047 (3)	
H27	0.4975	0.0639	1.0437	0.057*	
C14	0.5868 (4)	0.0264 (15)	0.6776 (18)	0.049 (3)	
C13	0.5692 (3)	0.1717 (12)	0.6255 (14)	0.038 (2)	
H23	0.5812	0.2205	0.5260	0.045*	
C15	0.5738 (4)	−0.0695 (14)	0.819 (2)	0.060 (4)	
H25	0.5880	−0.1648	0.8353	0.072*	
C18	0.6213 (5)	−0.0337 (18)	0.559 (2)	0.082 (4)	
H18	0.6343	−0.1202	0.6275	0.099*	
C16	0.5434 (5)	−0.0469 (13)	0.941 (2)	0.066 (4)	
H26	0.5410	−0.1266	1.0291	0.079*	
C8	0.2724 (4)	0.483 (2)	0.668 (2)	0.087 (4)	
H8	0.2534	0.4871	0.5679	0.104*	
C9A	0.2598 (7)	0.613 (3)	0.800 (4)	0.087 (4)	0.69 (4)
H9A	0.2623	0.7168	0.7426	0.130*	0.69 (4)
H9B	0.2764	0.6094	0.9055	0.130*	0.69 (4)
H9C	0.2327	0.5959	0.8346	0.130*	0.69 (4)
C10A	0.2694 (7)	0.314 (2)	0.747 (5)	0.087 (4)	0.69 (4)
H10A	0.2774	0.2367	0.6564	0.130*	0.69 (4)
H10B	0.2425	0.2926	0.7818	0.130*	0.69 (4)
H10C	0.2864	0.3050	0.8507	0.130*	0.69 (4)
C9B	0.2709 (15)	0.656 (4)	0.742 (9)	0.087 (4)	0.31 (4)
H9D	0.2819	0.7287	0.6532	0.130*	0.31 (4)
H9E	0.2861	0.6628	0.8518	0.130*	0.31 (4)
H9F	0.2440	0.6857	0.7662	0.130*	0.31 (4)
C10B	0.2558 (15)	0.367 (6)	0.810 (6)	0.087 (4)	0.31 (4)
H10D	0.2287	0.3943	0.8354	0.130*	0.31 (4)
H10E	0.2711	0.3744	0.9193	0.130*	0.31 (4)
H10F	0.2570	0.2583	0.7641	0.130*	0.31 (4)
C19A	0.6081 (9)	−0.107 (5)	0.383 (4)	0.082 (4)	0.60 (6)
H19A	0.5888	−0.1893	0.4069	0.123*	0.60 (6)
H19B	0.6305	−0.1531	0.3222	0.123*	0.60 (6)
H19C	0.5965	−0.0245	0.3082	0.123*	0.60 (6)
C20A	0.6523 (9)	0.101 (3)	0.534 (6)	0.082 (4)	0.60 (6)
H20A	0.6595	0.1435	0.6509	0.123*	0.60 (6)
H20B	0.6414	0.1851	0.4605	0.123*	0.60 (6)
H20C	0.6754	0.0568	0.4763	0.123*	0.60 (6)
C19B	0.6116 (14)	−0.039 (8)	0.356 (4)	0.082 (4)	0.40 (6)
H19D	0.5866	−0.0922	0.3390	0.123*	0.40 (6)
H19E	0.6319	−0.0975	0.2934	0.123*	0.40 (6)
H19F	0.6101	0.0689	0.3098	0.123*	0.40 (6)
C20B	0.6584 (9)	0.057 (6)	0.603 (8)	0.082 (4)	0.40 (6)
H20D	0.6632	0.0523	0.7310	0.123*	0.40 (6)
H20E	0.6555	0.1676	0.5660	0.123*	0.40 (6)
H20F	0.6802	0.0093	0.5391	0.123*	0.40 (6)

### Atomic displacement parameters (Å^2^)

**Table:**

	*U*^11^	*U*^22^	*U*^33^	*U*^12^	*U*^13^	*U*^23^
Pb1	0.04021 (16)	0.03651 (14)	0.02070 (12)	0.00435 (15)	−0.0009 (6)	0.0007 (4)
O1	0.044 (4)	0.055 (4)	0.026 (3)	0.003 (4)	0.001 (3)	−0.008 (3)
O11	0.046 (4)	0.035 (3)	0.033 (3)	0.003 (3)	0.005 (3)	0.008 (3)
O12	0.058 (5)	0.042 (4)	0.016 (3)	0.013 (3)	0.004 (3)	0.008 (3)
C6	0.069 (7)	0.072 (6)	0.013 (9)	−0.003 (5)	−0.007 (5)	−0.001 (5)
C7	0.069 (8)	0.049 (5)	0.023 (5)	−0.003 (5)	−0.007 (5)	−0.002 (4)
C1	0.055 (6)	0.030 (4)	0.027 (5)	0.003 (5)	−0.004 (5)	−0.001 (4)
C2	0.052 (7)	0.049 (6)	0.021 (5)	0.002 (5)	−0.007 (5)	0.000 (4)
C12	0.037 (6)	0.019 (4)	0.033 (4)	0.001 (4)	0.004 (4)	0.003 (3)
C11	0.052 (7)	0.028 (4)	0.022 (4)	−0.011 (4)	0.001 (4)	−0.001 (4)
C3	0.039 (6)	0.076 (8)	0.035 (5)	−0.005 (6)	0.006 (5)	−0.009 (6)
C5	0.054 (8)	0.063 (8)	0.070 (10)	−0.003 (6)	−0.030 (8)	0.006 (7)
C4	0.059 (9)	0.079 (9)	0.057 (9)	0.006 (7)	−0.007 (7)	−0.012 (8)
O2	0.049 (4)	0.096 (6)	0.017 (6)	−0.004 (4)	−0.001 (3)	−0.012 (4)
C17	0.073 (8)	0.037 (5)	0.032 (5)	−0.010 (6)	−0.001 (5)	0.005 (4)
C14	0.060 (8)	0.038 (6)	0.050 (7)	0.013 (6)	−0.009 (6)	−0.003 (5)
C13	0.050 (6)	0.040 (5)	0.023 (4)	0.005 (4)	0.001 (4)	0.006 (4)
C15	0.069 (7)	0.038 (5)	0.072 (11)	0.013 (6)	0.004 (7)	0.014 (6)
C18	0.070 (7)	0.078 (8)	0.098 (10)	0.019 (6)	0.023 (7)	−0.003 (7)
C16	0.115 (13)	0.035 (6)	0.047 (7)	0.004 (6)	0.018 (8)	0.014 (5)
C8	0.042 (6)	0.142 (11)	0.077 (10)	0.005 (6)	0.002 (6)	0.005 (9)
C9A	0.042 (6)	0.142 (11)	0.077 (10)	0.005 (6)	0.002 (6)	0.005 (9)
C10A	0.042 (6)	0.142 (11)	0.077 (10)	0.005 (6)	0.002 (6)	0.005 (9)
C9B	0.042 (6)	0.142 (11)	0.077 (10)	0.005 (6)	0.002 (6)	0.005 (9)
C10B	0.042 (6)	0.142 (11)	0.077 (10)	0.005 (6)	0.002 (6)	0.005 (9)
C19A	0.070 (7)	0.078 (8)	0.098 (10)	0.019 (6)	0.023 (7)	−0.003 (7)
C20A	0.070 (7)	0.078 (8)	0.098 (10)	0.019 (6)	0.023 (7)	−0.003 (7)
C19B	0.070 (7)	0.078 (8)	0.098 (10)	0.019 (6)	0.023 (7)	−0.003 (7)
C20B	0.070 (7)	0.078 (8)	0.098 (10)	0.019 (6)	0.023 (7)	−0.003 (7)

### Geometric parameters (Å, °)

**Table:**

Pb1—O11	2.327 (6)	C18—C20B	1.495 (18)
Pb1—O12	2.420 (7)	C18—C19A	1.496 (18)
Pb1—O1	2.422 (7)	C18—C19B	1.529 (19)
Pb1—O2	2.479 (9)	C18—C20A	1.539 (17)
Pb1—O12^i^	2.625 (7)	C18—H18	0.9800
Pb1—O1^i^	3.016 (8)	C16—H26	0.9300
Pb1—O11^ii^	3.064 (8)	C8—C9A	1.509 (16)
O1—C1	1.278 (14)	C8—C10A	1.518 (17)
O11—C11	1.293 (12)	C8—C10B	1.526 (19)
O12—C12	1.297 (11)	C8—C9B	1.534 (19)
O12—Pb1^ii^	2.626 (7)	C8—H8	0.9800
C6—C5	1.35 (2)	C9A—H9A	0.9600
C6—C7	1.398 (17)	C9A—H9B	0.9600
C6—H6	0.9300	C9A—H9C	0.9600
C7—C1	1.419 (15)	C10A—H10A	0.9600
C7—H7	0.9300	C10A—H10B	0.9600
C1—C2	1.488 (15)	C10A—H10C	0.9600
C2—O2	1.237 (13)	C9B—H9D	0.9600
C2—C3	1.419 (17)	C9B—H9E	0.9600
C12—C13	1.418 (14)	C9B—H9F	0.9600
C12—C11	1.432 (13)	C10B—H10D	0.9600
C11—C17	1.416 (14)	C10B—H10E	0.9600
C3—C4	1.372 (18)	C10B—H10F	0.9600
C3—H3	0.9300	C19A—H19A	0.9600
C5—C4	1.43 (2)	C19A—H19B	0.9600
C5—H5	0.9300	C19A—H19C	0.9600
C4—C8	1.52 (2)	C20A—H20A	0.9600
C17—C16	1.375 (17)	C20A—H20B	0.9600
C17—H27	0.9300	C20A—H20C	0.9600
C14—C15	1.384 (17)	C19B—H19D	0.9600
C14—C13	1.396 (15)	C19B—H19E	0.9600
C14—C18	1.538 (19)	C19B—H19F	0.9600
C13—H23	0.9300	C20B—H20D	0.9600
C15—C16	1.37 (2)	C20B—H20E	0.9600
C15—H25	0.9300	C20B—H20F	0.9600
			
O11—Pb1—O12	67.3 (2)	C14—C18—H18	106.5
O11—Pb1—O1	94.6 (3)	C20A—C18—H18	106.5
O12—Pb1—O1	76.2 (3)	C15—C16—C17	129.7 (12)
O11—Pb1—O2	83.2 (3)	C15—C16—H26	115.1
O12—Pb1—O2	128.0 (3)	C17—C16—H26	115.1
O1—Pb1—O2	64.1 (2)	C9A—C8—C10A	113.4 (15)
O11—Pb1—O12^i^	72.0 (2)	C9A—C8—C4	120.7 (16)
O12—Pb1—O12^i^	126.99 (19)	C10A—C8—C4	103.7 (15)
O1—Pb1—O12^i^	140.3 (3)	C9A—C8—C10B	85 (2)
O2—Pb1—O12^i^	77.0 (2)	C4—C8—C10B	129 (2)
C1—O1—Pb1	120.3 (6)	C10A—C8—C9B	137 (3)
C11—O11—Pb1	119.4 (6)	C4—C8—C9B	99 (2)
C12—O12—Pb1	115.9 (6)	C10B—C8—C9B	109.8 (18)
C12—O12—Pb1^ii^	128.3 (6)	C9A—C8—H8	106.0
Pb1—O12—Pb1^ii^	107.0 (3)	C10A—C8—H8	106.0
C5—C6—C7	136.4 (13)	C4—C8—H8	106.0
C5—C6—H6	111.8	C10B—C8—H8	107.3
C7—C6—H6	111.8	C9B—C8—H8	102.3
C6—C7—C1	127.1 (12)	C8—C9A—H9A	109.5
C6—C7—H7	116.5	C8—C9A—H9B	109.5
C1—C7—H7	116.5	H9A—C9A—H9B	109.5
O1—C1—C7	118.5 (10)	C8—C9A—H9C	109.5
O1—C1—C2	117.0 (9)	H9A—C9A—H9C	109.5
C7—C1—C2	124.5 (11)	H9B—C9A—H9C	109.5
O2—C2—C3	119.6 (11)	C8—C10A—H10A	109.5
O2—C2—C1	115.5 (10)	C8—C10A—H10B	109.5
C3—C2—C1	124.9 (11)	H10A—C10A—H10B	109.5
O12—C12—C13	115.0 (9)	C8—C10A—H10C	109.5
O12—C12—C11	117.1 (9)	H10A—C10A—H10C	109.5
C13—C12—C11	127.8 (9)	H10B—C10A—H10C	109.5
O11—C11—C17	117.7 (9)	C8—C9B—H9D	109.5
O11—C11—C12	117.9 (9)	C8—C9B—H9E	109.5
C17—C11—C12	124.3 (10)	H9D—C9B—H9E	109.5
C4—C3—C2	136.5 (12)	C8—C9B—H9F	109.5
C4—C3—H3	111.7	H9D—C9B—H9F	109.5
C2—C3—H3	111.7	H9E—C9B—H9F	109.5
C6—C5—C4	126.2 (13)	C8—C10B—H10D	109.5
C6—C5—H5	116.9	C8—C10B—H10E	109.5
C4—C5—H5	116.9	H10D—C10B—H10E	109.5
C3—C4—C5	123.7 (15)	C8—C10B—H10F	109.5
C3—C4—C8	119.3 (14)	H10D—C10B—H10F	109.5
C5—C4—C8	116.9 (14)	H10E—C10B—H10F	109.5
C2—O2—Pb1	121.1 (7)	C18—C19A—H19A	109.5
C16—C17—C11	130.6 (12)	C18—C19A—H19B	109.5
C16—C17—H27	114.7	H19A—C19A—H19B	109.5
C11—C17—H27	114.7	C18—C19A—H19C	109.5
C15—C14—C13	124.5 (12)	H19A—C19A—H19C	109.5
C15—C14—C18	118.9 (11)	H19B—C19A—H19C	109.5
C13—C14—C18	116.5 (12)	C18—C20A—H20A	109.5
C14—C13—C12	132.3 (10)	C18—C20A—H20B	109.5
C14—C13—H23	113.8	H20A—C20A—H20B	109.5
C12—C13—H23	113.8	C18—C20A—H20C	109.5
C16—C15—C14	130.6 (11)	H20A—C20A—H20C	109.5
C16—C15—H25	114.7	H20B—C20A—H20C	109.5
C14—C15—H25	114.7	C18—C19B—H19D	109.5
C20B—C18—C19A	130 (2)	C18—C19B—H19E	109.5
C20B—C18—C19B	113.9 (17)	H19D—C19B—H19E	109.5
C20B—C18—C14	110 (2)	C18—C19B—H19F	109.5
C19A—C18—C14	113.4 (16)	H19D—C19B—H19F	109.5
C19B—C18—C14	114 (2)	H19E—C19B—H19F	109.5
C19A—C18—C20A	113.1 (15)	C18—C20B—H20D	109.5
C19B—C18—C20A	93 (2)	C18—C20B—H20E	109.5
C14—C18—C20A	110.4 (15)	H20D—C20B—H20E	109.5
C20B—C18—H18	83.2	C18—C20B—H20F	109.5
C19A—C18—H18	106.5	H20D—C20B—H20F	109.5
C19B—C18—H18	125.3	H20E—C20B—H20F	109.5
			
O11—Pb1—O1—C1	92.9 (8)	C7—C6—C5—C4	2(3)
O12—Pb1—O1—C1	158.1 (8)	C2—C3—C4—C5	−1(3)
O2—Pb1—O1—C1	12.7 (7)	C2—C3—C4—C8	−179.4 (16)
O12^i^—Pb1—O1—C1	25.7 (10)	C6—C5—C4—C3	3(3)
O12—Pb1—O11—C11	11.0 (7)	C6—C5—C4—C8	−178.6 (15)
O1—Pb1—O11—C11	84.0 (7)	C3—C2—O2—Pb1	−176.8 (9)
O2—Pb1—O11—C11	147.2 (7)	C1—C2—O2—Pb1	6.5 (14)
O12^i^—Pb1—O11—C11	−134.3 (7)	O11—Pb1—O2—C2	−108.3 (10)
O11—Pb1—O12—C12	−13.3 (7)	O12—Pb1—O2—C2	−54.3 (10)
O1—Pb1—O12—C12	−114.3 (7)	O1—Pb1—O2—C2	−9.8 (9)
O2—Pb1—O12—C12	−73.9 (8)	O12^i^—Pb1—O2—C2	178.6 (10)
O12^i^—Pb1—O12—C12	29.5 (7)	O11—C11—C17—C16	−176.5 (13)
O11—Pb1—O12—Pb1^ii^	137.0 (4)	C12—C11—C17—C16	0.7 (19)
O1—Pb1—O12—Pb1^ii^	36.0 (3)	C15—C14—C13—C12	1(2)
O2—Pb1—O12—Pb1^ii^	76.4 (4)	C18—C14—C13—C12	177.0 (12)
O12^i^—Pb1—O12—Pb1^ii^	179.8 (3)	O12—C12—C13—C14	−178.3 (12)
C5—C6—C7—C1	−2(2)	C11—C12—C13—C14	−1(2)
Pb1—O1—C1—C7	163.2 (7)	C13—C14—C15—C16	−2(2)
Pb1—O1—C1—C2	−14.9 (12)	C18—C14—C15—C16	−178.0 (16)
C6—C7—C1—O1	176.5 (11)	C15—C14—C18—C20B	−106 (3)
C6—C7—C1—C2	−5.5 (19)	C13—C14—C18—C20B	78 (3)
O1—C1—C2—O2	5.4 (16)	C15—C14—C18—C19A	100 (2)
C7—C1—C2—O2	−172.6 (11)	C13—C14—C18—C19A	−76 (2)
O1—C1—C2—C3	−171.2 (11)	C15—C14—C18—C19B	125 (3)
C7—C1—C2—C3	10.9 (18)	C13—C14—C18—C19B	−51 (3)
Pb1—O12—C12—C13	−167.3 (7)	C15—C14—C18—C20A	−132 (2)
Pb1^ii^—O12—C12—C13	49.7 (13)	C13—C14—C18—C20A	52 (3)
Pb1—O12—C12—C11	14.6 (11)	C14—C15—C16—C17	3(3)
Pb1^ii^—O12—C12—C11	−128.3 (8)	C11—C17—C16—C15	−2(3)
Pb1—O11—C11—C17	169.3 (7)	C3—C4—C8—C9A	51 (2)
Pb1—O11—C11—C12	−8.1 (11)	C5—C4—C8—C9A	−127 (2)
O12—C12—C11—O11	−5.0 (13)	C3—C4—C8—C10A	−77 (2)
C13—C12—C11—O11	177.3 (10)	C5—C4—C8—C10A	104 (2)
O12—C12—C11—C17	177.9 (10)	C3—C4—C8—C10B	−59 (4)
C13—C12—C11—C17	0.1 (16)	C5—C4—C8—C10B	122 (3)
O2—C2—C3—C4	176.4 (17)	C3—C4—C8—C9B	66 (3)
C1—C2—C3—C4	−7(3)	C5—C4—C8—C9B	−113 (3)

Symmetry codes: (i) −*x*+1, −*y*+1, *z*+1/2; (ii) −*x*+1, −*y*+1, *z*−1/2.

## References

[bb1] Abrahams, I., Choi, N., Hendrick, K., Joyce, H., Matthews, R. W. & McPartlin, M. (1994). *Polyhedron*, **13**, 513–516.

[bb2] Barret, M. C., Mahon, M. F., Molloy, K. C., Steed, J. W. & Wright, P. (2001). *Inorg. Chem.* **40**, 4384–4388. 10.1021/ic0100368 11487346

[bb3] Barret, M. C., Mahon, M. F., Molloy, K. C. & Wright, P. (2000). *Main Group Met. Chem.* **23**, 663–671.

[bb4] Barret, M. C., Mahon, M. F., Molloy, K. C., Wright, P. & Creeth, J. E. (2002). *Polyhedron*, **21**, 1761–1766.

[bb5] Bondi, A. (1964). *J. Phys. Chem.* **68**, 441–451.

[bb6] Harrowfield, J. M., Maghaminia, S. & Soudi, A. A. (2004). *Inorg. Chem.* **43**, 1810–1812. 10.1021/ic034835x 15018492

[bb7] Ho, D. M. (2010). *Acta Cryst.* C**66**, m145–m148. 10.1107/S0108270110015581 20522935

[bb8] Kuma (1996). *KM-4 Software* Kuma Diffraction Ltd, Wrocław, Poland.

[bb9] Kuma (2001). *DATAPROC* Kuma Diffraction Ltd, Wrocław, Poland.

[bb10] Lyczko, K., Narbutt, J., Paluchowska, B., Maurin, J. K. & Persson, I. (2006). *Dalton Trans.* pp. 3972–3976. 10.1039/b606543k 17028705

[bb11] Lyczko, K., Starosta, W. & Persson, I. (2007). *Inorg. Chem.* **46**, 4402–4410. 10.1021/ic061561f 17474735

[bb12] Malik, M. A., O’Brien, P., Motevalli, M., Jones, A. C. & Leedham, T. (1999). *Polyhedron*, **18**, 1641–1646.

[bb13] Nomiya, K., Onodera, K., Tsukagoshi, K., Shimada, K., Yoshizawa, A., Itoyanagi, T., Sugie, A., Tsuruta, S., Sato, R. & Kasuga, N. Ch. (2009). *Inorg. Chim. Acta*, **362**, 43–55.

[bb14] Nomiya, K., Yoshizawa, A., Tsukagoshi, K., Kasuga, N. C., Hirakawa, S. & Watanabe, J. (2004). *J. Inorg. Biochem.* **98**, 46–60. 10.1016/j.jinorgbio.2003.07.002 14659632

[bb15] Oxford Diffraction (2000). *CrysAlis RED* Oxford Diffraction Ltd, Abingdon, England.

[bb16] Sheldrick, G. M. (2008). *Acta Cryst.* A**64**, 112–122. 10.1107/S0108767307043930 18156677

[bb17] Shimoni-Livny, L., Glusker, J. P. & Bock, Ch. W. (1998). *Inorg. Chem.* **37**, 1853–1867.

